# Audio-Based Emotion Recognition Using Self-Supervised Learning on an Engineered Feature Space

**DOI:** 10.3390/ai5010011

**Published:** 2024-01-17

**Authors:** Peranut Nimitsurachat, Peter Washington

**Affiliations:** 1 Institute for Computational and Mathematical Engineering (ICME), Stanford University, Stanford, CA 94305, USA; 2 Information and Computer Sciences, University of Hawai’i at Mānoa, Honolulu, HI 96822, USA

**Keywords:** emotion classification, emotion recognition, self-supervised learning, transfer learning

## Abstract

Emotion recognition models using audio input data can enable the development of interactive systems with applications in mental healthcare, marketing, gaming, and social media analysis. While the field of affective computing using audio data is rich, a major barrier to achieve consistently high-performance models is the paucity of available training labels. Self-supervised learning (SSL) is a family of methods which can learn despite a scarcity of supervised labels by predicting properties of the data itself. To understand the utility of self-supervised learning for audio-based emotion recognition, we have applied self-supervised learning pre-training to the classification of emotions from the CMU Multimodal Opinion Sentiment and Emotion Intensity (CMU- MOSEI)’s acoustic data. Unlike prior papers that have experimented with raw acoustic data, our technique has been applied to encoded acoustic data with 74 parameters of distinctive audio features at discrete timesteps. Our model is first pre-trained to uncover the randomly masked timestamps of the acoustic data. The pre-trained model is then fine-tuned using a small sample of annotated data. The performance of the final model is then evaluated via overall mean absolute error (MAE), mean absolute error (MAE) per emotion, overall four-class accuracy, and four-class accuracy per emotion. These metrics are compared against a baseline deep learning model with an identical backbone architecture. We find that self-supervised learning consistently improves the performance of the model across all metrics, especially when the number of annotated data points in the fine-tuning step is small. Furthermore, we quantify the behaviors of the self-supervised model and its convergence as the amount of annotated data increases. This work characterizes the utility of self-supervised learning for affective computing, demonstrating that self-supervised learning is most useful when the number of training examples is small and that the effect is most pronounced for emotions which are easier to classify such as happy, sad, and angry. This work further demonstrates that self-supervised learning still improves performance when applied to the embedded feature representations rather than the traditional approach of pre-training on the raw input space.

## 1. Introduction

Emotion classification has become increasingly used in a multitude of academic disciplines, including digital psychiatry [1,2], autonomous vehicles [3,4], and media analytics [5,6]. By having sufficient labeled training data, researchers can improve the performance of emotion classifiers. A disproportionate amount of prior work in emotion recognition with video data places a stronger emphasis on the visual modality and often neglects the acoustic modality. Thus, deep learning models that classify emotions from audio input can potentially improve the performance of existing emotion classifiers.

A major challenge of training a performant emotion classifier for both the visual and acoustic modalities is the paucity of annotated data. Deep learning models are usually trained using a supervised-learning paradigm in which the model learns to map inputs—such as speech, facial movement, or facial expression—to the output or corresponding emotions. In order to attain a high performance, the model usually requires a large annotated training set; this type of dataset, however, tends to be scarce. The lack of sufficient annotated data, therefore, makes training deep learning models to classify emotions accurately challenging. One solution is to manually annotate the unlabeled dataset with corresponding emotions; however, this task is very demanding and requires well-trained annotators.

In this paper, we propose a self-supervision technique which can boost the accuracy of a deep learning models by taking advantage of additional pre-training steps on unannotated embedded audio data. We conduct our experiment on the CMU-MOSEI dataset [7]—one of the largest multimodal datasets to date. We apply a methodology similar to the pre-training of large language models [8], where we pre-train our model on acoustic data before fine-tuning it on the small amount of annotated emotion data. By pre-training the model on the unlabeled data, we discover that it can acquire valuable internal representations of the embedded acoustic data which, in turn, can increase the model’s accuracy in the fine-tuning stage, even with a small number of annotated data. This work further demonstrates that self-supervised learning (SSL) is effective when applied to the embedding space rather than the input space.

## 2. Related Work

Self-supervised learning has been applied mostly in natural language processing and computer vision. For instance, the Bidirectional Encoder Representations from Transformers (BERT) [8] model pre-trains deep bi-directional representations on a large corpus through masked language modeling and next sentence prediction. Masked language modeling involves simply masking some percentage of the input tokens at random and predicting those masked tokens [8]. The pre-trained model is then transferred to a fine-tuning step where it is fine-tuned using annotated data. This pre-training technique significantly improves the model’s overall performance, especially when the number of labels is too small for deep learning.

Recent works have proposed using similar self-supervised techniques on audio in a raw format in order to perform several tasks such as speech recognition or emotion classification. In the case of emotion classification, Audio-only Self-Supervision (Odd) [9] jumbles 25% of the clips; two windows of a length of 15% of the selected clips are randomly selected and swapped. The encoder is then tasked to identify the elements in the input batch that have been swapped. During this pre-training step, the model will learn a useful representation of the audio which can be beneficial in classifying discrete emotions in the fine-tuning step. The Odd model explores this pre-training technique on several datasets, including CREMA-D [10], Ravdess [11], and IEMOCAP [12]. All of these datasets store audio in raw format. Large language models like GPT-3 and GPT-4 [13] also use a similar strategy to predict the following word in unlabeled text during the pre-training step.

Self-supervised techniques have also been applied to other models such as CPC (Contrast Predictive Coding) [14] and APC (Autoregressive Predictive Coding) [15]. In the case of CPC (Contrast Predictive Coding), the authors propose a universal unsupervised learning approach to extract useful representations from high-dimensional data such as audio data. This framework pre-trains the model by making predictions of the next token given the previous tokens. Specifically, it tries to learn representations that separate the target future frame from randomly sampled negative frames. This pre-trained model is then fine-tuned and evaluated on phone prediction performance. CPC (Contrast Predictive Coding) [14] is trained on the publicly available LibriSpeech dataset [16] and takes in audio inputs as 16KHz PCM audio waveforms. Similary to CPC (Contrast Predictive Coding) [14], APC (Autoregressive Predictive Coding) [15] also tries to predict the target future frames within the audio input. However, unlike CPC (Contrast Predictive Coding) [14], which focuses on separating a target frame from randomly sampled negative frames, APC is only allowed to ignore information that is common across the dataset. This APC framework [15] takes in audio data in the form of an 80-dimensional log Mel spectogram.

Another relevant work is the Problem Agnostic Speech Encoder (PASE) [17]. This method pre-trains the model by predicting the seven extracted features—Waveform, Log power spectrum (LPS), Mel-frequency cepstral coefficients (MFCC), Prosody, Local info max (LIM), Global info max (GIM), and Sequence predicting coding (SPC)—of raw audio. This pre-trained model can be used for any downstream tasks by adding the linear classifier on top of the frozen pre-trained layer. Finally, the SeCoSt framework [18] also applied a teacher–student self-supervised approach to audio by using the predictions of the current iteration as labels for the next one. Similarly to previous works, the input features are in the form of logmel features.

As discussed above, most, if not all, of the previous works on self-supervised audio apply this technique on the raw audio input. Our proposed method, however, will apply the masking technique to the encoded input data. Furthermore, we also monitor the performance of this technique for each of the six emotions, in addition to the overall performance across all emotions. We are unaware of prior studies in the literature that characterize this discrepancy.

## 3. Methods

### 3.1. Dataset and Features

**Figure 1.**
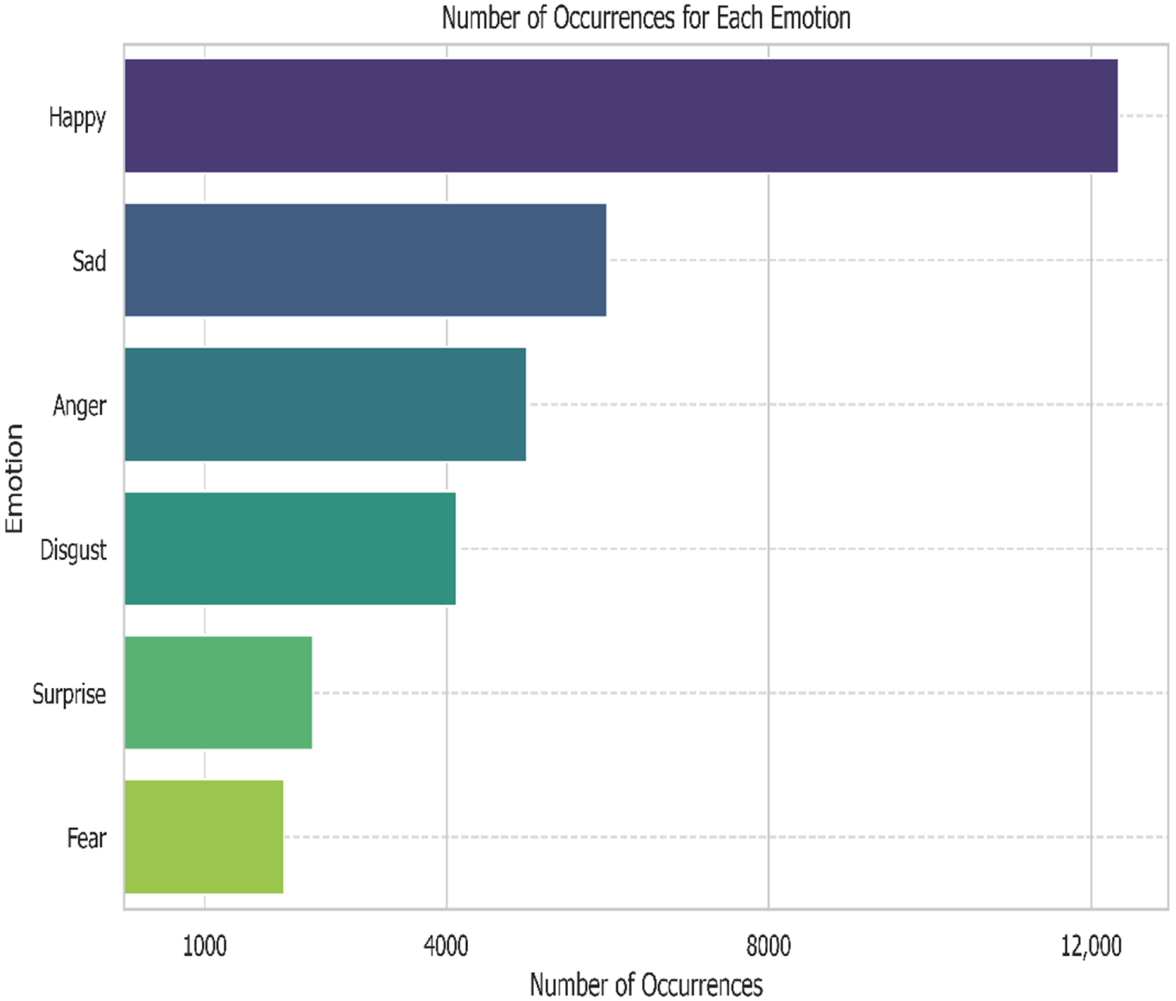
Distribution of emotions in CMU-MOSEI.

The experiment was conducted on CMU Multimodal Opinion Sentiment and Emotion Intensity (CMU-MOSEI) [7], which is considered to be one of the largest datasets of multimodal sentiment analysis and emotion recognition to date. This dataset contains more than 23,500 sentence utterance videos from 1000 speakers on YouTube and has three modalities, including acoustic and visual. The dataset is also annotated with labels that indicate the level of six Ekman emotions [19], including happiness, sadness, anger, surprise, disgust, and fear. The labels are an array of six numbers; each one represents each of the intensities of each emotion f on a scale from 0 to 3: [0: no evidence of e, 1: weakly f, 2: e, 3: highly f] [20]. These annotations are assigned by 3 crowdsourced judges from the Amazon Mechanical Turk platform who received prior training and received a 98% or higher [20] approval rate to assure high quality annotations. The distribution of emotions in this dataset is shown in Figure 1.

Here, we work with the emotion labels and the acoustic modality of CMU-MOSEI. It is important to note that the acoustic data in CMU-MOSEI are not provided in a raw format; instead, each timestep of the input consists of 74 parameters that are extracted from COVAREP software v1.0.1 [21]. These 74 parameters can be categorized into 5 groups: Periodicity and Synchronisation, Sinusoidal Modeling, Spectral Envelope Estimation and Formant Tracking, Glottal Analysis, and Phase Processing. The variables in each of these groups represent different characteristics of the original audio. For instance, the variables in Periodicity and Synchronisation include F0, VUV, and creak; they contain information about the estimation of fundamental frequency from the speech signal, the speech polarity (asymmetric excitation signal at the glottis), and the identification of the Glottal Closure Instants, respectively [21]. Some of the 74 features have been used as input parameters in prior machine learning models for emotion or sentiment classifications. However, none of these models has incorporated self-supervised learning. We provide one of the first explorations of self-supervised learning on a dataset that is encoded with a pre-established feature embedding.

### 3.2. Model Details

We pre-trained a deep learning model to uncover the randomly masked timesteps within the audio clips. As mentioned in the previous section, each timestep of the audio input consisted of 74 parameters extracted from COVAREP software v1.0.1 [21].

**Figure 2.**
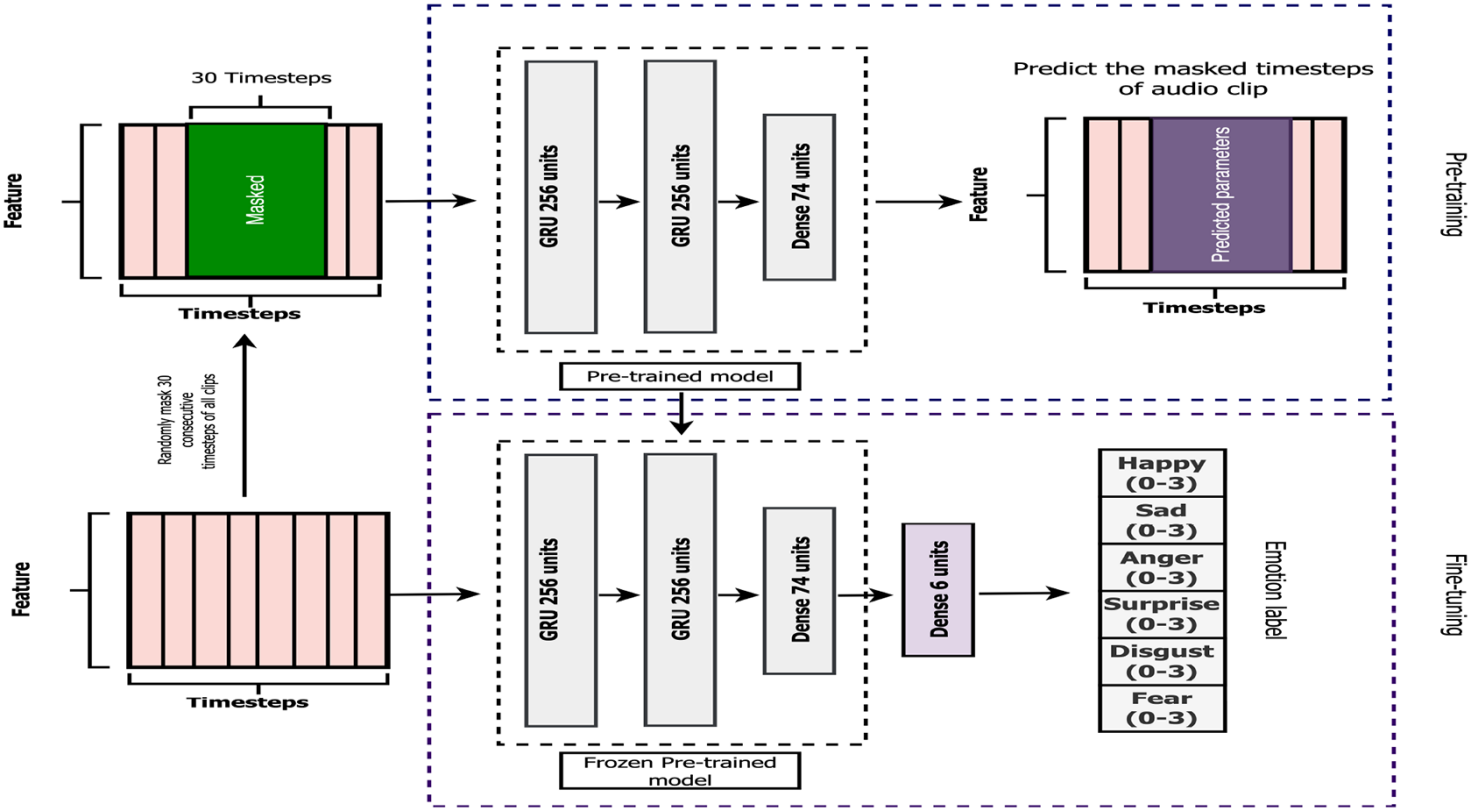
An overview of our proposed methodology for pre-training the deep learning model before transferring the pre-trained model to the fine-tuning step.

Approximately 10% of the audio input or 30 consecutive timesteps were randomly selected from every input; we randomly chose a valid starting timestep, ensuring that the subsequent 30 timesteps fell within the given input range. As shown in Figure 2, all 74 parameters of the selected timesteps were masked or replaced with −30, which is completely outside of the standardized range of each parameter. During our self-supervised pre-training step, the masked version of the original audio was used as an input variable for our model, while the original version of the input was a target variable. Therefore, the input and output of the pre-training step had the same dimension (74 × number of timesteps in the audio), as depicted in Figure 2. Our deep learning model, consisting of two layers of a 256-unit GRU followed by one 74-unit dense layer, was then trained during this self-supervised pre-training step to predict the features of the original audio clip given the generated clip with masked features. In other words, the model was pre-trained to uncover the masked 30 consecutive timesteps within the original audio clip without using any emotion labels.

By pre-training the model in this way, we encouraged the pre-trained model to learn useful representations of the parameters of the audio clip that can be transferred to the fine-tuning step where the pre-trained model was fine-tuned with a small number of inputs annotated with emotion labels.

As shown in the bottom block of Figure 2, the pre-trained model from the first step was then transferred to the fine-tuning step. We introduced a single 6-unit dense layer on top of the pre-trained model to enable the new model to predict the emotion label from the audio input. This label is represented as an array of 6 floating-point numbers. The 6-unit dense layer was then fine-tuned with a small fraction of audio input and corresponding emotion labels, while all layers of the pre-trained model were frozen. This implies that the parameters of the pre-trained model from the initial step remained unchanged throughout the fine-tuning process, with only the additional parameters of the 6-unit dense layer being fine-tuned to classify emotions. The input and output of this fine-tuning step were the original audio input with no masked timesteps (74 × number of timesteps in the audio) and emotion labels (an array of 6 values), respectively. The performance of the final model after the fine-tuning step was evaluated on the unseen validation set and compared with that of the baseline model with an identical architecture.

### 3.3. Experiment

We explored the performance of our deep learning model pre-trained with self-supervised learning in comparison to the baseline model with a identical architecture. The performance of the model was evaluated through the overall mean absolute error (MAE), mean absolute error (MAE) for each emotion, 4-class overall accuracy, and 4-class accuracy for each of six emotions. We selected these two metrics because they are commonly used by other state-of-the-art models to evaluate their performances on CMU-MOSEI. Considering that each of the 6 values in the emotion label was a floating-point number rather than an integer, direct accuracy calculation became challenging. Hence, utilizing 4-class accuracy was more appropriate for this dataset, as it involves rounding each float to the nearest integer before computing the accuracy.


(1) r(x)={⌊x⌋ifx−⌊x⌋<0.5⌈x⌉ifx−⌊x⌋≥0.5}

Given the rounding function in Equation (1), the 4-class overall accuracy is defined as follows. The metrics in Equation (2) consist of six columns that represent the predicted and true values of six emotions of e samples.


(2) $$f_{4-class}(\left[\begin{array}{ccc} x_{11}^{pred} & \ldots & x_{16}^{pred}\\ x_{21}^{pred} & \ldots & x_{26}^{pred}\\ \ldots & \; & \;\\ x_{n1}^{pred} & \ldots & x_{n6}^{pred} \end{array}\right],\left[\begin{array}{ccc} x_{11}^{true} & \ldots & x_{16}^{true}\\ x_{21}^{true} & \ldots & x_{26}^{true}\\ \ldots & \; & \;\\ x_{n1}^{true} & \ldots & x_{n6}^{true} \end{array}\right])\;\;=\frac{\sum_{k=1}^{n}\sum_{i=1}^{6}1[r(x_{ki}^{pred})=r(x_{ki}^{true})]}{6n}$$

The 4-class accuracy for each emotion is defined similarly. However, instead of considering all six columns of six emotions, only one column that corresponds to the emotion of interest is considered.


(3) $$f_{4-class}^{i}(\left[\begin{array}{ccc} x_{11}^{pred} & \ldots & x_{16}^{pred}\\ x_{21}^{pred} & \ldots & x_{26}^{pred}\\ \ldots & \; & \;\\ x_{n1}^{pred} & \ldots & x_{n6}^{pred} \end{array}\right],\left[\begin{array}{ccc} x_{11}^{true} & \ldots & x_{16}^{true}\\ x_{21}^{true} & \ldots & x_{26}^{true}\\ \ldots & \; & \;\\ x_{n1}^{true} & \ldots & x_{n6}^{true} \end{array}\right])\;\;=\frac{\sum_{k=1}^{n}1[r(x_{kj}^{pred})=r(x_{kj}^{true})]}{n}\;\text{when}\;0\leq j\leq 6$$

The overall mean absolute error (MAE) for each emotion use the standard definition of MAE on all six columns and one column of interest, respectively.

**Figure 3.**
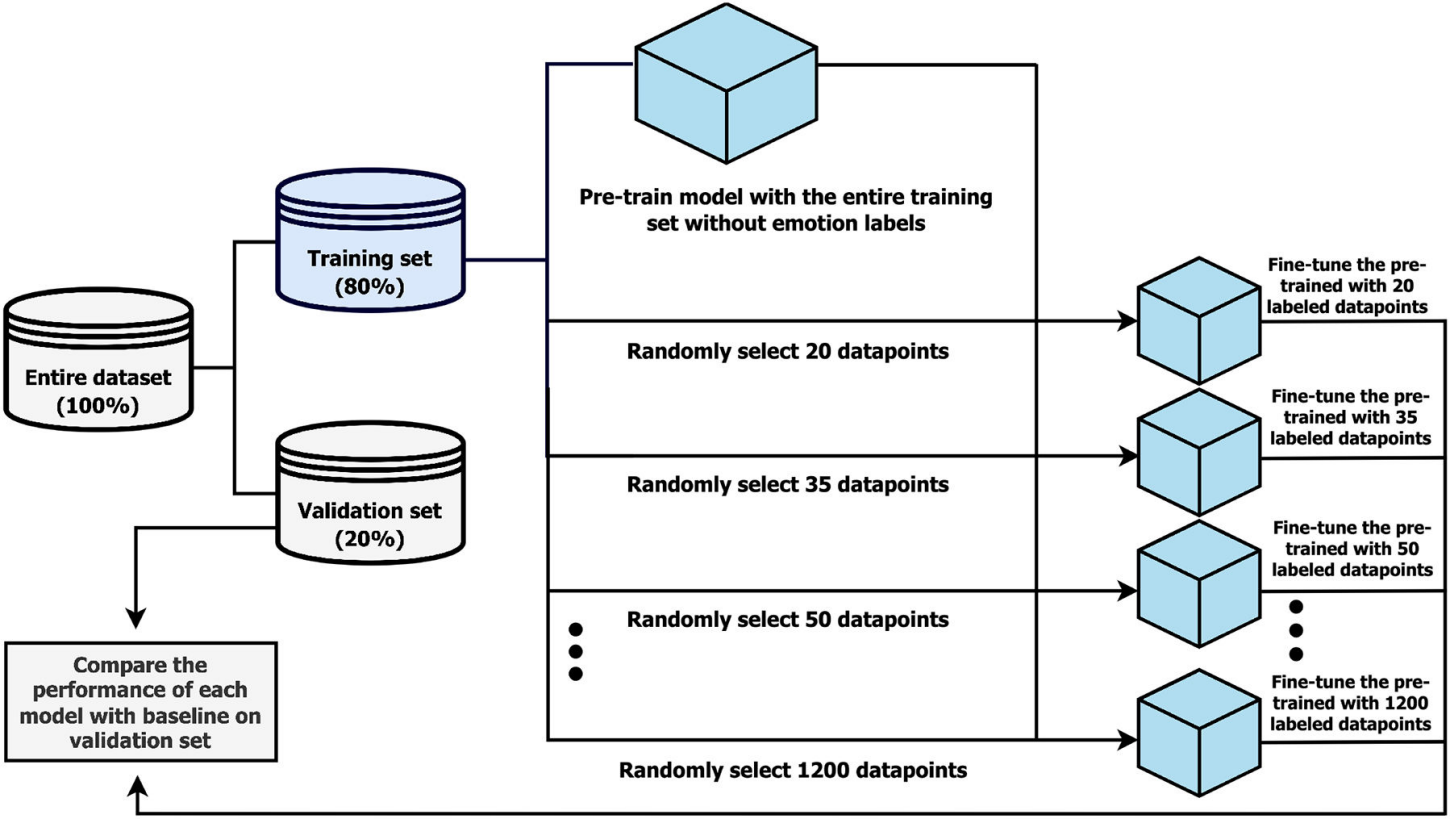
An overview of our experiment on how to pre-train and fine-tune the model with a variety of numbers of annotated datapoints and evaluate the performance of models in comparison to the baseline.

As shown in Figure 3, the entire dataset was split into a training set (80%) and validation set (20%). The training set was used to train the baseline model and self-supervised models; the baseline model had the same architecture (two layers of 256-unit GRU and one 74-unit dense layer, one layer of 74-unit dense, and one layer of 6-unit dense) as the final pre-trained model shown in Figure 2, but had no pre-training. These two models were evaluated on the evaluation set using the four metrics mentioned above. All audio inputs in the training set were used to pre-train the model without any emotion labels. We masked all 74 parameters of 30 randomly selected consecutive timesteps and trained a deep neural network model with two layers of 256-unit GRUs and one 6-unit dense layer to uncover these masked timesteps, as discussed in the previous section. By predicting the masked timesteps of the original audio input, the pre-trained model learned useful representation that could be transferred to the downstream task. It is imperative to note that we did not use the emotion labels of the audio input at all in the pre-training step. This pre-trained model was then fine-tuned to the emotion labels only in the fine-tuning step. As shown in Figure 3, we fine-tuned our pre-trained model with different numbers of annotated data points in order to monitor the benefit of our pre-training technique when annotated data are scarce. Additionally, we aim to monitor the convergence of our pre-trained model’s performance to the baseline. This analysis will provide valuable insights into the settings where our method is the most useful. During the fine-tuning step, each data point represented the pair of the original audio input in an encoded form and its corresponding emotion label. In this experiment, we monitored the performance of the pre-trained and baseline models at 20, 35, 50, … , 200 and 400, 600, … , and 1200 data points. This amount of labeled data is very small compared to the available labels we have in CMU-MOSEI.

A sample of data points were randomly selected from the training dataset, along with their corresponding emotion labels; these annotated data points were then used to fine-tune the pre-trained model and train the baseline model for 30 epochs. We used Adam as the optimizer, with its default learning rate of 0.001 and Mean Squared Error (MSE) as the loss function. The baseline and pre-trained models were then evaluated on the unseen evaluation set according to four performance metrics. We performed these three steps—randomly sampling, fine-tuning, and evaluating–three times per each number of data points (20, 35, 50) in order to monitor the mean and variance of evaluation metrics.

It is imperative to note that this paper primarily focuses on the advantages of self-supervised learning when the amount of available labeled data is small and traditional methods of training supervised models are not effective. Therefore, emphasis is placed on evaluating the performance of our pre-trained model against the baseline in that setting (20–1200 annotated data points). This makes it challenging to compare our results directly to those from other state-of-the-art models that utilize most, if not all, of the labels in CMU-MOSEI for fine-tuning. In order to compare our results to those from other state-of-the-art models, we calculated an additional metric, the F1-score, at our maximum labeled data quantity (1200). We select F1 since it is one of the most commonly used metrics on this dataset. Our calculated F1, at our maximum labeled data quantity (1200), is compared to the F1-score from other state-of-the-art models with much larger labeled training sets.

## 4. Results

**Figure 4.**
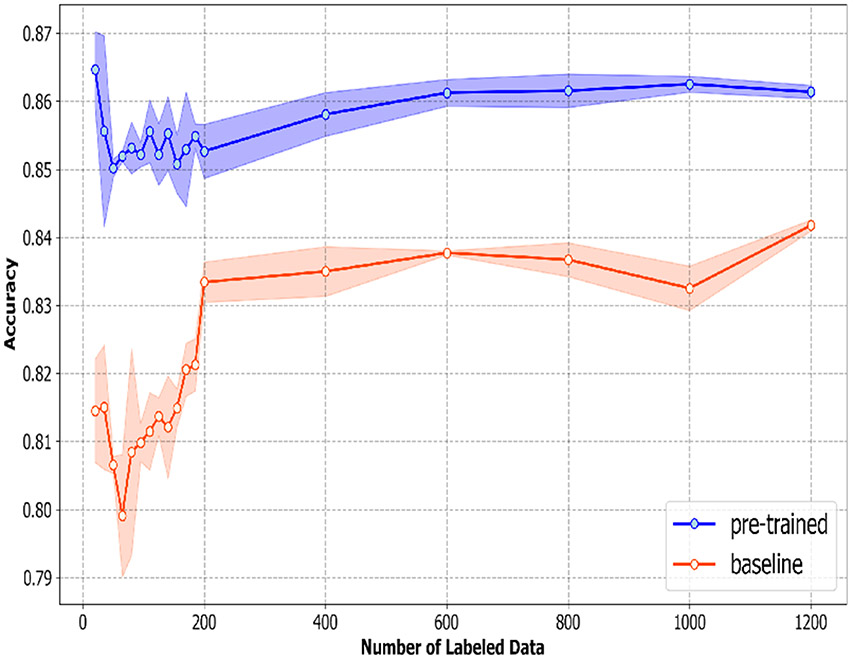
The 4-class overall accuracy across all six emotions (Happiness, Sadness, Anger, Surprise, Disgust, Fear) along with the standard deviation calculated from three bootstrapped samples of the labeled data.

Figure 4 shows the four-class overall accuracy, along with the standard deviation of the baseline and pre-trained model at each amount of labeled data; the standard deviation in the figure is calculated from the accuracy of three iterations at each amount of labeled data. The four-class overall accuracy of the pre-trained model is around 85–87% when there are a few labeled data (0–200). The accuracy gradually increases as the number of labeled data increases. The accuracy of the baseline model, on the other hand, starts at around 81–82% and increases rapidly as the number of labeled data approaches 200. According to Figure 4, it is clear that the pre-trained model outperforms the baseline around 4–5% with a small number of labeled data; the four-class overall accuracy of both models starts to converge as the number of labeled data increases. It is also interesting to note that, for both baseline and pre-trained models, the standard deviations are relatively high with few labeled data and become smaller once the number of labeled data increases.

**Figure 5.**
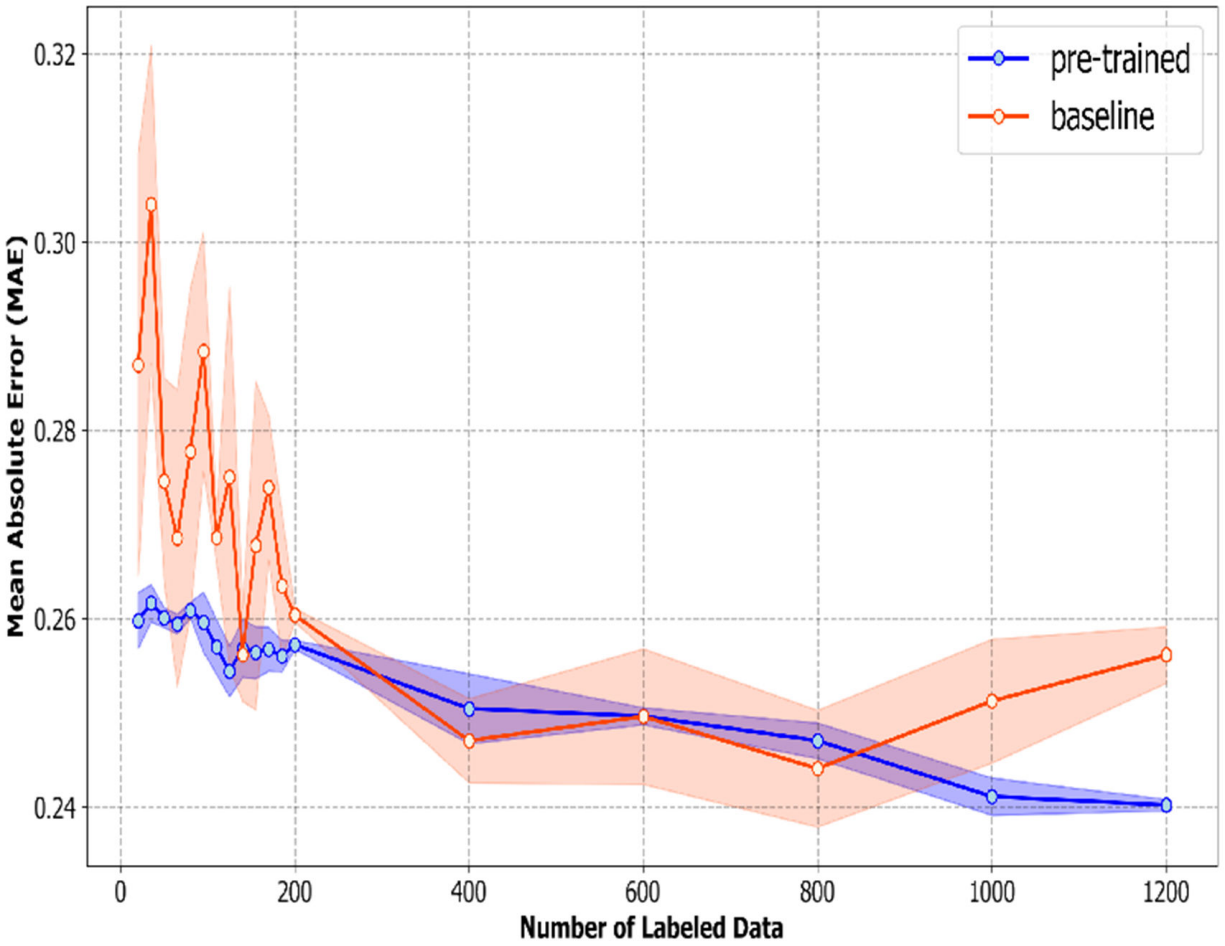
The MAE across all six emotions (Happiness, Sadness, Anger, Surprise, Disgust, Fear) along with the standard deviation calculated from three bootstrapped samples of the labeled data.

Similarly, the overall mean absolute error (MAE) of the pre-trained model is around 0.26 at 20 labeled data and continues to shrink as the number of labeled data increases. The mean absolute error of the baseline model, however, is around 0.29–0.30 at 20 labeled data and shrinks as the number of labeled data increases. As shown in Figure 5, the pre-trained model consistently outperforms the baseline, especially when the number of labeled data is small. However, the discrepancy between the overall mean absolute error (MAE) of pre-trained and baseline models diminishes as the quantity of labeled data increases. At our maximum labeled data quantity (1200), the difference in mean absolute error (MAE) between our pre-trained model and the baseline is less than 0.02, which is significantly narrower than those observed at lower numbers of annotated data. The lower Mean Absolute Error (MAE) observed in the pre-trained model compared to the baseline across all numbers of labeled data emphasizes the advantages of self-supervised learning (SSL), especially in situations with limited available labeled data. This is because, even with the largest labeled data size (1200), the quantity of labeled data that we used for fine-tuning (1200) remains exceptionally small in relation to the CMU-MOSEI dataset or any training datasets for emotion classification tasks. This shows that our method works well when there are plenty of unlabeled data and only a few labeled examples—a situation we often encounter in the real world. Apart from the higher accuracy and lower mean absolute error, the standard deviations of these two metrics of the pre-trained model are also lower than the baseline model. This indicates that our pre-trained model can outperform the baseline model not only in accuracy but also stability.

**Figure 6.**
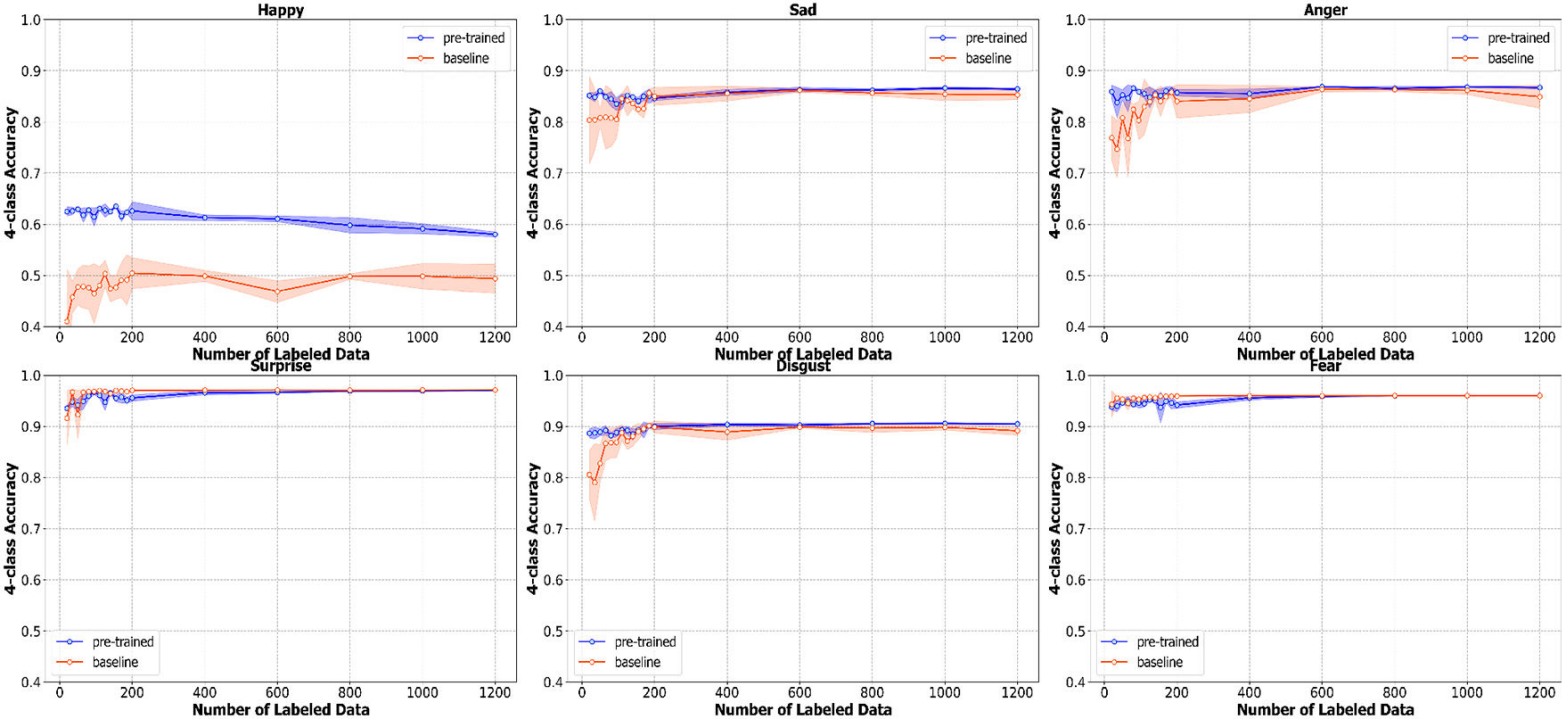
The 4-class accuracy for each of six emotions (Happiness, Sadness, Anger, Surprise, Disgust, Fear) along with the standard deviation calculated from three bootstrapped samples of the labeled data.

As mentioned in the previous section, we also monitor the mean absolute error and four-class accuracy for each emotion (happiness, sadness, anger, surprise, disgust, and fear). According to Figure 6, the pre-trained model outperforms the baseline in the accuracy of happiness, sadness, anger, and disgust by a large margin. The largest gap between the accuracy of pre-trained and baseline models can be seen in happiness and anger, in which the pre-trained model outperforms by approximately 20% and 10%, respectively. Our pre-trained technique, however, does not improve the accuracy of emotions like surprise and fear. Figure 6 shows that the baseline model performs slightly better than the pre-trained model. This can stem from the fact that these two emotions are relatively uncommon in this dataset, according to the distribution in Figure 1. Thus, the pre-trained model might not be able to benefit from the large amount of unlabeled data in the pre-training step. Furthermore, the accuracy of these two emotions is relatively high at around 90%; this could potentially make it difficult to improve beyond this threshold. The reason we visualize all metrics at these small numbers of labeled data is to show that all metrics start to converge as the number of labeled data increases. Thus, the effect of our method is most prominent in a situation with a very small amount of labeled data and becomes less obvious as the amount of labeled data increases. For example, the pre-trained model outperforms the baseline with a larger margin at 20 compared to at 1200 labeled data points.

**Figure 7.**
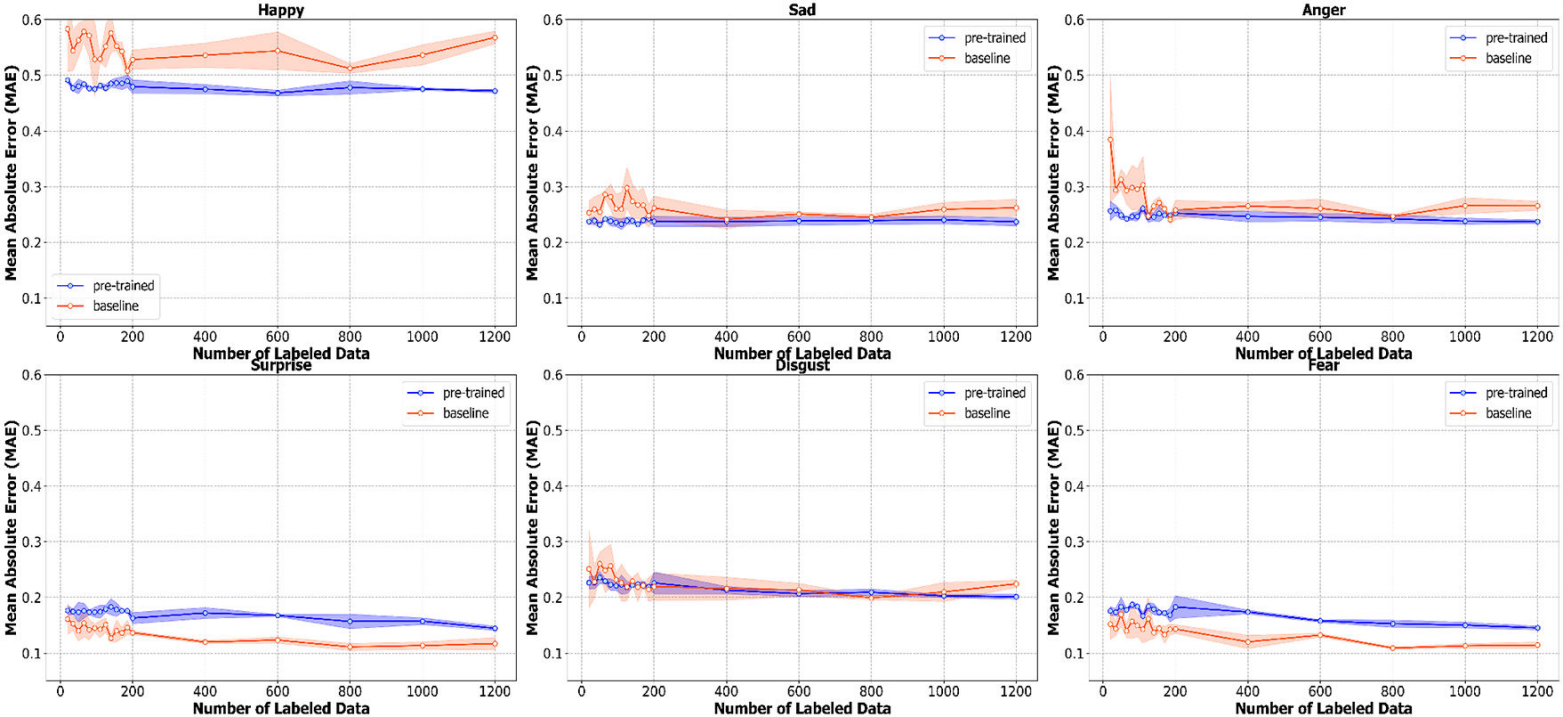
A plot showing the mean absolute error (MAE) for each of six emotions (Happiness, Sadness, Anger, Surprise, Disgust, Fear) along with the standard deviation calculated from three bootstrapped samples of the labeled data.

The mean absolute error for each emotion in Figure 7 also shows similar results. Firstly, we can see the large gap in the mean absolute error of the baseline and pre-trained models in most emotions when the number of labels is small; this large gap then starts to shrink as the number of labels increases. The pre-trained model also outperforms the baseline with a large margin in traditionally “easier” emotions (e.g., happiness, sadness, and anger). The benefit of our pre-training technique, however, becomes marginal when coping with more nuanced expressions (e.g., surprise and fear). As discussed in the case of four-class accuracy, the pre-trained model might not be able to benefit from the the large amount of unlabeled data in the pre-training step when classifying more nuanced expressions due to the scarcity of these emotions in the dataset. Furthermore, the mean absolute error of these nuanced expressions is low at approximately 0.12–0.16 compared to the mean absolute error of traditionally “easier” emotions, which can be as high as 0.4–0.5. Therefore, it might be difficult for our pre-trained model to improve beyond this.

In addition to four-class accuracy and MAE, we also calculate the F1 scores of our pre-trained model (SSL) at our maximum labeled data quantity (1200) for each of six emotion labels: happiness, sadness, anger, surprise, disgust, and fear. These values are compared to F1 scores from other state-of-the-art models, including RF, SVM, DAN, DHN, Adieu-Net, SER-LSTM, LSTM, SLSTM, BLSTM, SBLSTM, and RHN [22], which classify emotions from the acoustic modality in CMU-MOSEI. However, it is important to note that these state-of-the-art models have used the CMU-MOSEI Natural Split [22] to train the model; this split contains much more labeled data than our maximum data quantity (1200). The F1 scores derived from our pre-trained model are compared to those from BLSTM, a commonly-used model in CMU-MOSEI. Additionally, we compare our F1 scores to those obtained from the best-performing models in each emotion label, as reported in the prior literature (Best-Performing from the Prior Literature) [22]. Despite the limited size of our labeled training set, our SSL model outperforms BLSTM and achieves the highest F1 scores across all emotion labels among these state-of-the-art models, as shown in Table 1.

## 5. Discussion and Conclusions

Consistent with the prior self-supervised learning literature, we find that the benefits of self-supervised learning are observed when there are few supervised labels to learn from. Self-supervised learning provides a marginal benefit when the number of labels is medium-to-large. This can be seen in both four-class accuracy and mean absolute error; the pre-trained model outperforms the baseline when the number of labels is small (0–200) and gradually converges to the baseline as the number of labels increases. Our model also achieves higher F1 scores compared to other state-of-the-art models, despite the fact that it is fine-tuned with a smaller amount of labeled data. This confirms the practicality of our proposed pre-training method in real-world scenarios where the quantity of annotated data is typically much lower than that of unannotated data. Interestingly, we observe that the benefits of self-supervised learning are most pronounced for traditionally “easier” emotions (e.g., happiness, sadness, and anger) and more difficult for more nuanced expressions (e.g., surprise and fear). We are unaware of prior studies in the literature which characterize this discrepancy. Furthermore, related works on self-supervised learning usually apply this technique to raw audio data. This work, on the other hand, applies the self-supervised technique to the encoded audio data from COVAREP software v1.0.1 [21].

Although our pre-trained model can significantly outperform the baseline model in both evaluation metrics, there are some notable limitations to this work. Firstly, the baseline and pre-trained models are trained and evaluated for three iterations at each number of labels due to limited computing resources. We can obtain more stable results regarding the benefit of our pre-training technique by increasing the number of iterations at each number of labels. Furthermore, this work only applies the self-supervised technique to the audio modality. This could be another limitation, since this technique can also work with other available modalities within CMU-MOSEI [7] or even cross-modality classification, as discussed above.

Therefore, it would be interesting to investigate this pre-training technique on different encoded modalities within CMU-MOSEI [7] or datasets. Furthermore, we can also use the model pre-trained on an audio modality to guide emotion recognition as well as any similar tasks on other available modalities (e.g., visual). We also want to explore alternative architectures of deep neural networks (DNNs) that can potentially lead to a better performance in our pre-trained model. This pre-training technique can also be applied to the raw audio data, in which our results can be compared to the state-of-the-art methods from other related works.

On the other hand, the presented SSL methodology can be applied to datasets collected from video and audio data streams. While video-based emotion classifiers often use only visuals to make predictions, the addition of audio can bolster discriminative performance. These improved models can accelerate a variety of applications of affective computing. For example, *SuperpowerGlass* [23] is a wearable application that uses real-time computer vision to deliver real-time social cues to children with Autism Spectrum Disorder (ASD). The device uses the outward facing camera to read facial expressions of a conversation partner, and these expressions are classified into discrete Ekman emotions [19]. Nevertheless, a significant challenge in achieving a high-performing real-time emotion classification model on such devices is the limited availability of labeled data. Specifically, while such devices can collect a substantial volume of unlabeled data, such as facial expressions, acquiring the corresponding labels for this data can be challenging. This is precisely where our proposed technique becomes valuable, as it enables the model to leverage a substantial amount of unlabeled data, instead of solely relying on a limited set of labeled data. With our proposed technique, such AI-powered digital therapeutics can provide children with more real-time social cues reflecting the emotional expressions evoked by their conversation partners. *Guess What?,* another digital therapeutic for children with ASD, also curates videos enriched for emotion expression [24]. While these data have traditionally been used for purely visual prediction tasks [25], the addition of the audio modality combined with self-supervised learning can bolster performance to enable more personalized healthcare experiences. In general, self-supervised learning has the potential to learn the baseline temporal dynamics of data collected from a highly specialized domain distribution, such as in ASD diagnostics. These ‘personalized’ pre-trained models can be fine-tuned to traditionally complex target tasks like affect recognition.

## Figures and Tables

**Table 1.** Comparing F1 scores of self-supervised learning model (SSL) to other emotion classification models on acoustic modality. There are eleven models in total that we compare our SSL to: RF, SVM, DAN, DHN, Adieu-Net, SER-LSTM, LSTM, SLSTM, BLSTM, SBLSTM, and RHN.

Emotion	Result	
	Methodology	F1 Score
Happiness	SSL	0.77
	BLSTM	0.37
	Best-Performing from the Prior Literature	0.62
Sadness	SSL	0.93
	BLSTM	0.36
	Best-Performing from the Prior Literature	0.66
Anger	SSL	0.93
	BLSTM	0.16
	Best-Performing from the Prior Literature	0.72
Surprise	SSL	0.99
	BLSTM	0.74
	Best-Performing from the Prior Literature	0.74
Disgust	SSL	0.96
	BLSTM	0.22
	Best-Performing from the Prior Literature	0.76
Fear	SSL	0.98
	BLSTM	0.77
	Best-Performing from the Prior Literature	0.78

## Data Availability

CMU-MOSEI is the only dataset in this publication. It can be found at http://multicomp.cs.cmu.edu/resources/cmu-mosei-dataset/.
